# Navigating Diagnostic Dilemmas: Miller Fisher Syndrome in Pregnancy Masquerading As Stroke

**DOI:** 10.7759/cureus.98877

**Published:** 2025-12-10

**Authors:** Kumutha Nadaraj

**Affiliations:** 1 Internal Medicine, Universiti Kebangsaan Malaysia Medical Centre, Kuala Lumpur, MYS

**Keywords:** atypical gbs variant, miller fisher syndrome (mfs), ophthalmoplegia, pregnancy, rare neuroimmunological disorder

## Abstract

Miller Fisher Syndrome (MFS) is a rare variant of Guillain-Barré Syndrome (GBS) characterized by a clinical triad of acute ophthalmoplegia, ataxia, and areflexia. Despite being a rare, acquired demyelinating nerve disease, a higher incidence has been reported among the Asian population, where the incidence is estimated to be 18-26% of GBS compared to 3-5% among the Western population. An acute presentation of MFS can mimic a posterior circulation stroke. Other differential diagnosis includes Wernicke’s encephalopathy (WE), which is characterized by a clinical triad of ophthalmoplegia, nystagmus, and ataxia. In this case report, we describe a pregnant lady who presented with an acute onset of binocular diplopia, dizziness, nausea, and vomiting. This case highlights the importance of early recognition of MFS features, the diagnostic challenges associated with it, and the significance of prompt treatment in improving clinical outcomes.

## Introduction

Miller Fisher syndrome (MFS) is an acute immune-mediated demyelinating polyneuropathy, first described by James Collier in 1932 as a triad of ophthalmoplegia, ataxia, and areflexia [1]. In 1956, Charles Miller Fisher classified it as a distinct entity within the Guillain-Barré Syndrome (GBS) spectrum [2]. MFS arises from an aberrant autoimmune response, often triggered by preceding upper respiratory or gastrointestinal infections. Molecular mimicry leads to the production of antibodies against gangliosides in peripheral nerves, causing demyelination and the characteristic clinical features [3].

Epidemiologically, MFS is more common among Asian populations, accounting for 18-26% of GBS cases compared to 3-5% in Western populations [3,4]. Although contemporary population-based data in Malaysia are lacking, local studies suggest that while GBS occurs at rates comparable to global trends, MFS remains rare but documented [5].

Clinically, MFS can mimic a posterior circulation stroke due to the acute onset of ophthalmoplegia, ataxia, and associated nausea or vomiting. Pregnancy adds diagnostic complexity as physiological changes and pregnancy-associated symptoms can obscure classical neurological signs, and imaging modalities may be limited due to fetal safety considerations. Furthermore, in resource-limited settings, diagnosis often relies on detailed clinical assessment, basic laboratory tests, and cerebrospinal fluid analysis, rather than advanced neuroimaging or specialized antibody testing.

This case report highlights the presentation, diagnosis, and management of a pregnant woman with MFS in a rural setting with limited resources, emphasizing the diagnostic challenges and clinical considerations in such contexts.

## Case presentation

We report a 31-year-old Melanau lady, primigravida at 26 weeks of gestation, who presented to the Emergency Department with complaints of double vision, dizziness, nausea, and vomiting for 3 days. She reported that her double vision was sudden in onset, started upon waking up from sleep, and she had considerable difficulty walking due to dizziness and double vision. There was no diurnal variation in her symptoms. On further questioning, she admitted that she had an episode of upper respiratory infection about a week prior to the onset of symptoms. She otherwise denied any body weakness, sensory loss, speech difficulty, and bladder or bowel incontinence. Antenatally, she had gestational diabetes mellitus for which she was on diet control, and her blood sugar profile was well-controlled. On examination, she appeared uncomfortable and tended to keep her eyes closed. She was fully alert with a normal mental status examination. Her blood pressure was 125/73 mmHg, heart rate 82, saturation 100% under room air, and she was afebrile. She had a significant restriction of bilateral eye abduction and a mild restriction of bilateral eye adduction. She complained of having binocular diplopia at all gaze. Otherwise, there was no ptosis, proptosis, or nystagmus. Bilateral pupils were equal and reactive. The fundus examination showed a normal cup-to-disc ratio with no papilledema. Her facial strength and sensation were normal. The tone, muscle bulk, and power of all her limbs were normal. Other cranial nerve examinations were unremarkable. Her gait was stable when assessed with one eye closed (by eliminating diplopia). The fatiguability test was negative. The obstetric scan revealed a singleton pregnancy, which was at an appropriate gestational age.

**Figure 1:**
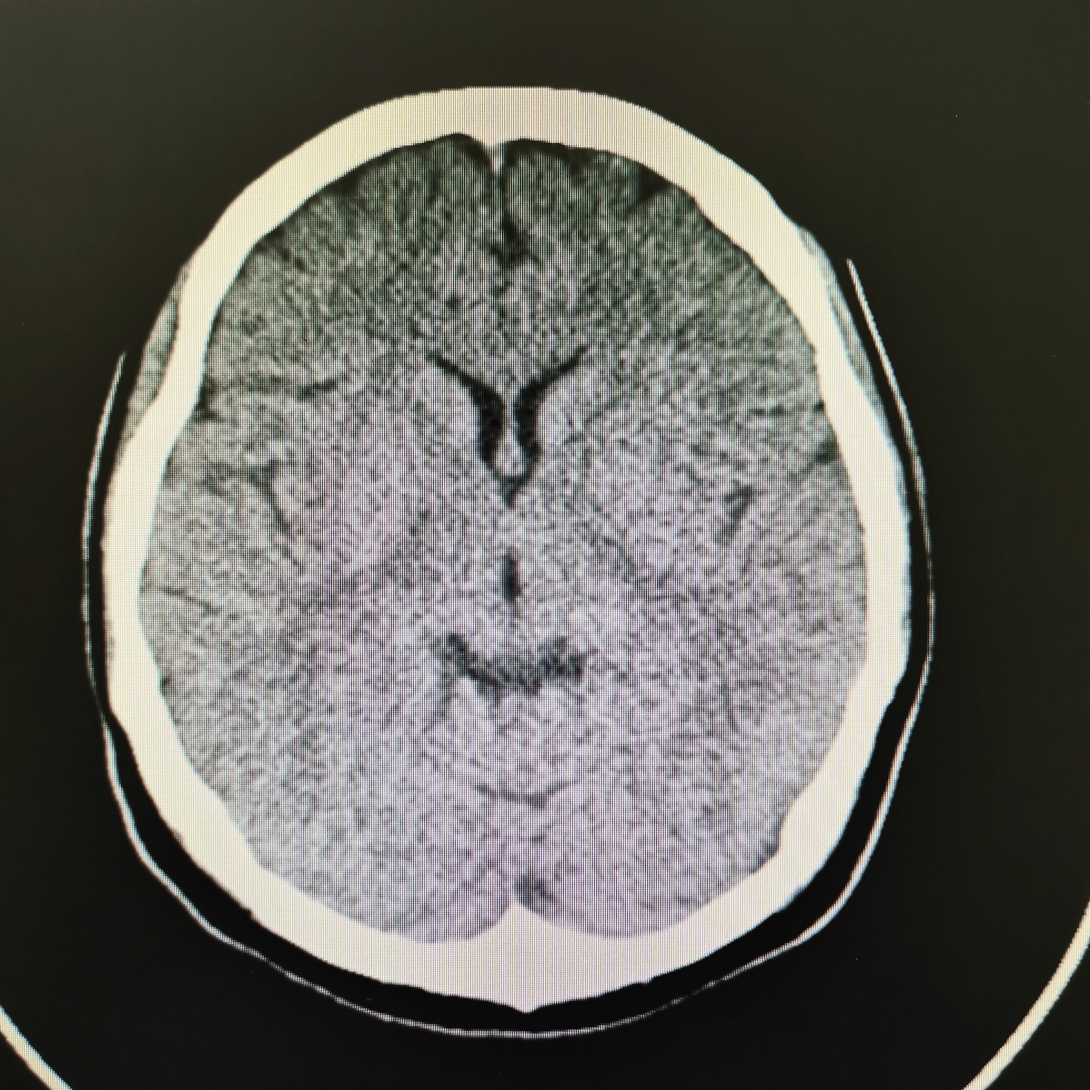
CT brain (axial view) shows no significant abnormalities

**Figure 2:**
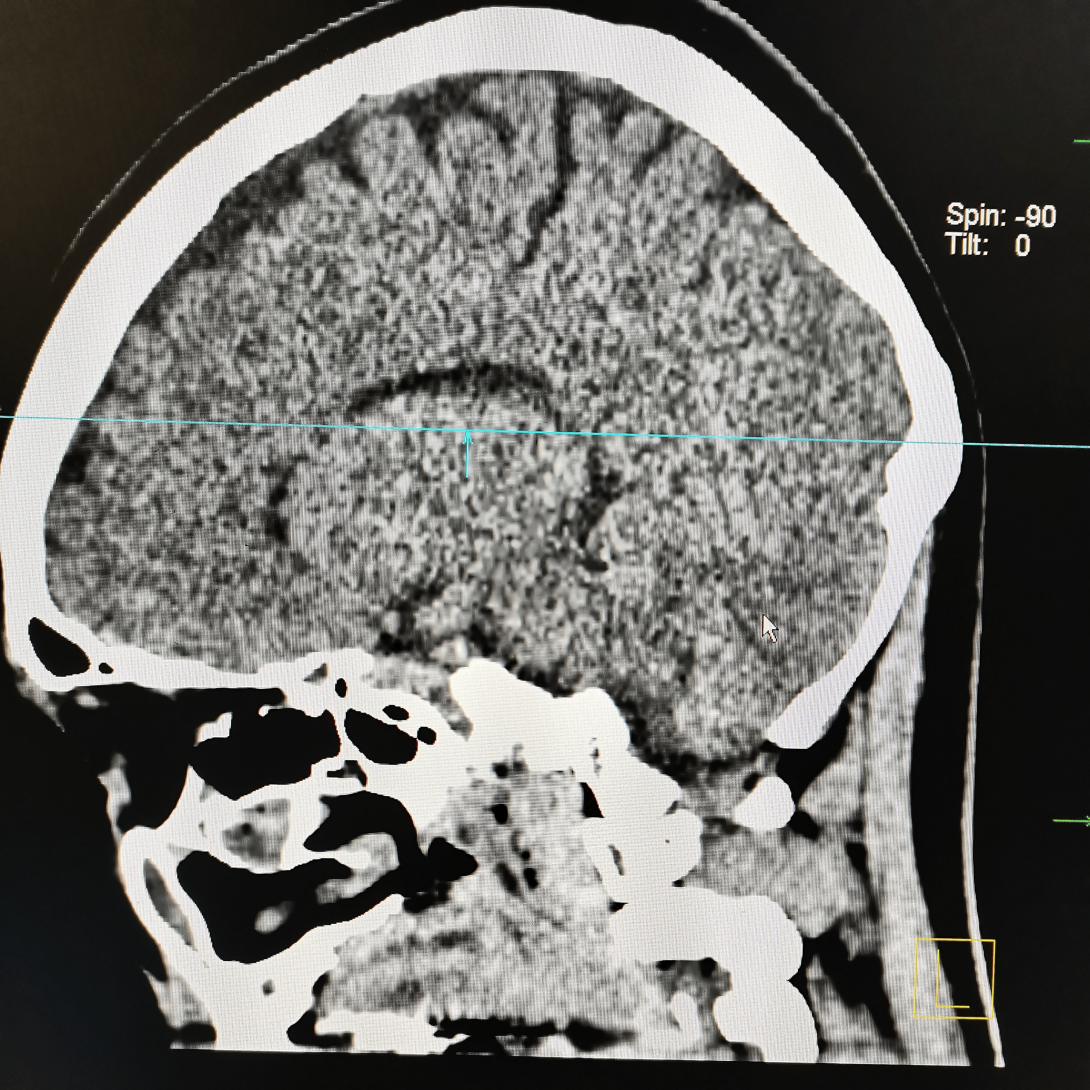
CT brain (sagittal view) shows no significant abnormalities

**Figure 3:**
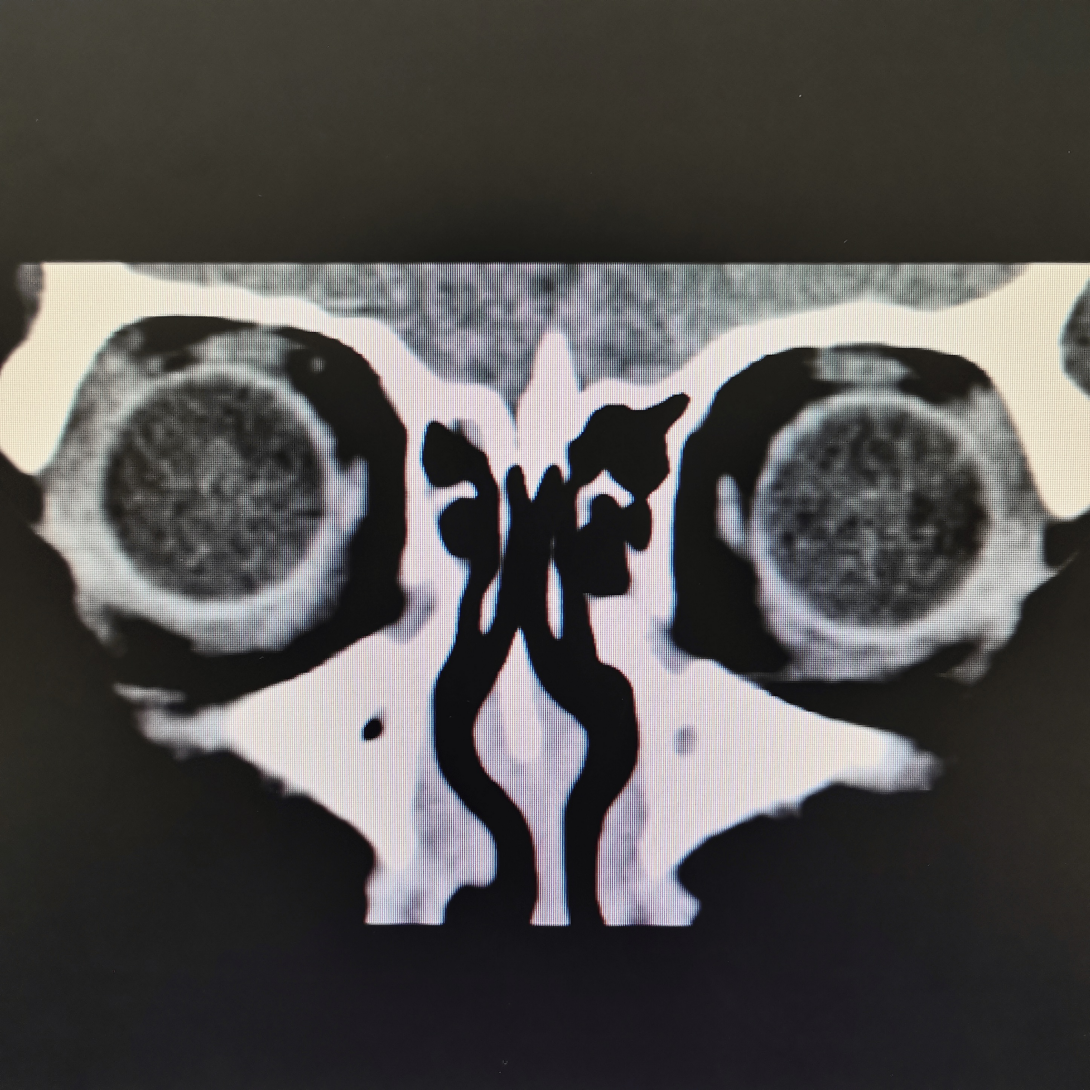
CT brain + ocular (coronal view) shows no significant abnormalities

At our center, an MRI was not available. The plain CT brain/orbit at 60 hours since the onset of the symptoms showed no significant abnormality, as demonstrated in Figures 1-3. Her full blood count (FBC), renal profile (RP), electrolytes, liver function test (LFT), and C-reactive protein (CRP) were within normal range. In addition, several serological studies were obtained, including a normal thyroid function test (TFT), negative rapid plasma regain (RPR), and HIV. Urine dipstick was negative for leucocytes, nitrite, and ketone. She was admitted for observation and further management. She was treated with intravenous thiamine. However, she didn’t show much clinical improvement. The following day, her ophthalmoplegia worsened, and she complained of muscle weakness in her bilateral shoulders and arms. Her neurological examination showed reduced muscle power over the bilateral upper limb, areflexia, and prominent ataxia. Notwithstanding her deteriorating ophthalmoplegia and neurological symptoms, her vital signs remained stable, and she was comfortable under room air.

After consultation with the neurology team for the course of disease progression, MFS became the primary working diagnosis. A lumbar puncture and cerebrospinal fluid (CSF) analysis on day 5 of illness demonstrated no remarkable findings except for an elevated CSF chloride level. The CSF analysis is as listed in Table 1. Nerve conduction studies and electromyography could not be conducted because these modalities were not accessible in our rural setting.

**Table 1:** CSF analysis

CSF Analysis	Result	Normal Range
Protein	0.26 g/L	0.01-0.45 g/L
Chloride	167 mmol/L	120-130 mmol/L
Glucose	3.5 mmol/L	2.5-4.5 mmol/L
WBC	2 cells/µL	0-5 cells/µL
Gram stain	Negative	-
AFB staining	Negative	-
Indian Ink	Negative	-
Culture and sensitivity	No growth	-
Meningitis panel	Negative	-

Her ophthalmoplegia continued to worsen throughout the admission, while the diplopia, dizziness, and upper limb weakness persisted. She was unable to ambulate due to her persistent dizziness. Subsequently, she was treated with a 5-day course of intravenous immunoglobulin (IVIG) on days 6-10 of illness. Upon completion of IVIG therapy, she claimed improvement in her bilateral upper limb muscle power. Power 5/5 in all four limbs. However, she had persistent diplopia with bilateral frozen eyes. Otherwise, her vital signs remained stable throughout the admission, and she had no other new complaints. She was then discharged home with vitamin B complex tablets and was advised to undergo physiotherapy and alternate eye patching.

At a follow-up visit, approximately 5 weeks after the onset of her symptoms, she had a stable gait with normal muscle power. She showed a noticeable improvement in her extraocular muscle movement. Her bilateral eye adduction improved by 50%, and eye elevation and depression showed 25% improvement, but she could still not abduct her eyes. During the follow-up visit at 3 months, she showed a complete resolution of ophthalmoplegia and had a stable gait.

## Discussion

MFS is primarily a clinical diagnosis characterized by the classical triad of acute ophthalmoplegia, ataxia, and areflexia [6]. However, various other clinical signs and symptoms have been reported, including facial, pupillary, bulbar muscle palsies, and neck and shoulder muscle weakness [6,7]. MFS often mimics other conditions such as posterior circulation stroke, Wernicke’s encephalopathy (WE), brainstem encephalitis, and myasthenia gravis (MG), which makes the diagnosis of MFS more challenging [1,4].

Our patient presented with an acute onset of ophthalmoplegia, which later progressed to ataxia and areflexia. Considering the hypercoagulable state in pregnancy, her initial symptoms of acute-onset diplopia with nausea and vomiting raised the suspicion of a posterior circulation stroke. Notably, MFS during pregnancy is exceedingly rare and presents a diagnostic dilemma due to overlapping symptoms with pregnancy-related conditions. The physiological changes of pregnancy can also obscure neurological signs. There is limited data in the literature describing MFS in the second trimester, making this case a valuable addition in expanding awareness of atypical neuro-immune presentations in pregnant patients.

In our patient, we proceeded with a CT scan instead of an MRI due to its unavailability. CT has well-recognized limitations for detecting acute posterior-circulation and brainstem infarcts. Diffusion-weighted MRI (DWI-MRI) is substantially more sensitive than non-contrast CT for acute ischemia and is considered the imaging standard for detecting small posterior-fossa and brainstem lesions. Studies report markedly higher infarct detection and diagnostic accuracy with DWI compared with CT, whereas non-contrast CT sensitivity for posterior-fossa ischemia has been reported as low (often <50% and in some series as low as 7-42%) [8]. Therefore, posterior circulation stroke remained the foremost consideration in the differential diagnosis for our patient.

Later on, her rapidly progressive ophthalmoplegia, symmetrical descending weakness, ataxia, and areflexia pointed clearly toward the diagnosis of MFS [2]. The diagnosis was further supported by the history of antecedent upper respiratory tract symptoms approximately one week prior to the onset of diplopia. Nearly two-thirds of MFS cases are preceded by upper respiratory or gastrointestinal infections that trigger an aberrant immune response, producing autoantibodies (notably anti-GQ1b) via molecular mimicry.

WE was another differential diagnosis considered, given her pregnancy, vomiting, and neurological presentation. However, the absence of nystagmus and the lack of clinical improvement following intravenous thiamine made this diagnosis less likely. MG was excluded based on the absence of ptosis, lack of diurnal fluctuation of symptoms, a negative fatiguability test, and no improvement with rest. Cavernous venous thrombosis (CVT), a recognized aseptic complication of pregnancy, was ruled out as well due to preserved visual fields, lack of ptosis or proptosis, absence of periorbital edema, normal facial sensation, and no papilledema on fundus examination [9].

A lumbar puncture and CSF analysis performed on day 5 of illness revealed unremarkable results, including normal cell counts and protein levels. Although our patient demonstrated an elevated CSF chloride level, this finding is not pathognomonic of MFS. CSF chloride is largely influenced by serum chloride and acid-base balance, and is well known to be affected by preanalytical and analytical factors such as laboratory methodology, sample handling, and evaporation in small-volume specimens. Notably, there is no evidence that pregnancy influences CSF chloride levels, and such an association has not been reported in the literature. The absence of cyto-albumin dissociation in this case is also consistent with early-stage MFS, where CSF protein may remain normal within the first week. Literature reported that albuminocytologic dissociation in CSF is only seen in 44-81% of cases of GBS and its variants [10]. Additionally, the diagnostic yield is lower in the early stages of illness, as in our case.

Anti-GQ1b antibodies are present in more than 85% of patients with MFS [11,12], and their levels have been shown to correlate with clinical severity, especially the extent of ophthalmoplegia [10]. These antibodies are generated due to molecular mimicry between microbial lipo-oligosaccharides (commonly from *Campylobacter jejuni* and *Haemophilus influenzae*) and the GQ1b ganglioside component of human cranial nerves. GQ1b is abundantly expressed in cranial nerves III, IV, and VI, muscle spindle afferent fibers, cerebellar granule cells, and dorsal root ganglia, explaining the clinical features of ophthalmoplegia, ataxia, and areflexia in MFS. This has been well demonstrated in a study by Chiba et al. [13]. Anti-GQ1b antibody testing was not performed in our patient due to resource limitations. Nonetheless, the classical clinical triad and progression pattern support the diagnosis. Notably, up to 10% of MFS cases may be anti-GQ1b antibody negative [7].

MFS is typically a self-limiting disorder with an excellent prognosis. Almost all patients recover fully within 6 months, regardless of specific treatment [14]. However, a Cochrane review of treatment options reported that intravenous immunoglobulin (IVIG) may hasten recovery in some patients [15]. Our patient received a course of IVIG, after which she showed full improvement in muscle strength and partial improvement in gait before discharge. The mean recovery time for MFS ranges from 8 to 12 weeks. Observational data suggest that ataxia typically resolves within 1 month, and ophthalmoplegia within 3 months [15]. In our patient, complete recovery of gait was noted by week 5, and full resolution of ophthalmoplegia by week 12.

## Conclusions

This case highlights the diagnostic challenge of MFS during pregnancy, where overlapping symptoms with obstetric conditions, limited resources, and atypical findings (such as normal early CSF or unexplained CSF chloride elevations) can delay diagnosis. It underscores the importance of maintaining a high index of suspicion for immune-mediated neuropathies in pregnant patients presenting with progressive cranial nerve involvement, and reinforces the value of clinical judgment in settings where diagnostic resources are constrained.

## References

[1] de Castillo LL, Diestro JD, Ignacio KH, Pasco PM (2019). A rare mimic of acute stroke: Rapidly progressing Miller-Fisher Syndrome to acute motor and sensory axonal neuropathy variant of Guillain-Barre Syndrome. BMJ Case Rep.

[2] Yepishin IV, Allison RZ, Kaminskas DA, Zagorski NM, Liow KK (2016). Miller Fisher Syndrome: A case report highlighting heterogeneity of clinical features and focused differential diagnosis. Hawaii J Med Public Health.

[3] Rocha Cabrero F, Morrison EH (2023). Miller Fisher Syndrome. https://www.ncbi.nlm.nih.gov/books/NBK507717/.

[4] Ono M, Sato H, Shirahashi M (2015). Clinical features of Miller-Fisher syndrome in pregnancy. Case Rep Obstet Gynecol.

[5] Tan CY, Razali SNO, Goh KJ, Shahrizaila N (2020). Diagnosis of Guillain-Barré syndrome and validation of the Brighton criteria in Malaysia. J Peripher Nerv Syst.

[6] Noorhayati Noorhayati, Puspha R, Premala Devi S (2021). Clinical case reports Miller Fisher syndrome variant: The incomplete triad. https://www.researchgate.net/profile/Puspha-Raman/publication/354208600_Clinical_Case_Reports_Case_Report_Miller_Fisher_Syndrome_Variant_The_Incomplete_Triad/links/612c80d22b40ec7d8bd1e240/Clinical-Case-Reports-Case-Report-Miller-Fisher-Syndrome-Variant-The-Incomplete-Triad.pdf.

[7] Ramakrishna KN, Tambe V, Kattamanchi A, Dhamoon AS (2020). Miller Fisher syndrome with bilateral vocal cord paralysis: A case report. J Med Case Rep.

[8] Hwang DY, Silva GS, Furie KL, Greer DM Comparative sensitivity of computed tomography vs. magnetic resonance imaging for detecting acute posterior fossa infarct. J Emerg Med.

[9] Liang ZW, Gao WL, Feng LM (2017). Clinical characteristics and prognosis of cerebral venous thrombosis in Chinese women during pregnancy and puerperium. Sci Rep.

[10] Rath J, Zulehner G, Schober B, Grisold A, Krenn M, Cetin H, Zimprich F (2021). Cerebrospinal fluid analysis in Guillain-Barré syndrome: Value of albumin quotients. J Neurol.

[11] Wang C, Wilson C, Thurtell M (2022). Miller Fisher syndrome 47-year-old male with sudden onset diplopia and bilateral ophthalmoplegia. EyeRounds.Org.

[12] Aljaafari D, Almustafa S, Saleh Ali A (2021). Miller Fisher variant of Guillain-Barré syndrome triggered by ventilator-associated pneumonia. Int Med Case Rep J.

[13] Chiba A, Kusunoki S, Obata H, Machinami R, Kanazawa I (1997). Ganglioside composition of the human cranial nerves, with special reference to pathophysiology of Miller Fisher syndrome. Brain Res.

[14] Bukhari S, Taboada J (2017). A case of Miller Fisher syndrome and literature review. Cureus.

[15] Overell JR, Hsieh ST, Odaka M, Yuki N, Willison HJ (2007). Treatment for Fisher syndrome, Bickerstaff's brainstem encephalitis and related disorders. Cochrane Database Syst Rev.

